# Notes from the Out-Patient Department

**Published:** 1888-03

**Authors:** W. J. Penny

**Affiliations:** Assistant-Surgeon to the Bristol General Hospital


### NOTES FROM THE OUT-PATIENT DEPARTMENT. By W. J. Penny, F.R.C.S., Assistant-Surgeon to the Bristol General Hospital.

*Patent Urachus.*—A. W., a healthy-looking child, æt. 11 months, came under observation in October, 1887.

The mother had borne eight children before this, all healthy and without deformity. At birth, the umbilical cord was noticed to be unduly large. It separated about the ninth day, and since that time a clear watery fluid has exuded from the umbilicus. At this part a raised round mass was to be seen, about the size of a hazel-nut. The surface was intensely red, and to all appearances covered with mucous membrane. A constriction existed at its junction with the abdomen. Surrounding the umbilicus was a dusky red areola, about one inch in width, evidently due to the constant irritation of the fluid. In the centre of the mass, a sinus existed, through which a thin watery fluid constantly exuded. A probe was passed into the sinus in a downward direction for five or six inches. On passing a catheter into the bladder, the two instruments could be made to touch each other. The child passed urine in the natural way; but in spite of that, and in a very short time after micturition, it again escaped from the allantoic fistula.

Operation was suggested, but declined by the mother.

### AFFECTIONS OF THE TONGUE.

*Brown Warty Growths on Tongue.*—J. F., æt. 42, French polisher, came to the hospital complaining of swelling of his knee, due to an accident a fortnight previously.

The patient had the earthy, emaciated, cachectic appearance, with thin brittle hair, characteristic of a constitution sodden with syphilis.

He admitted having had a chancre twenty years before, for which he was treated. He had no marked secondary symptoms. Three years after the attack he married. His wife was not infected, but did not become pregnant. At different periods during the last seventeen years he has had sores about him, which have yielded to treatment. On the upper part of the right arm, and on the left leg, were numerous circular scars. At the corners of his mouth were radiating scars. The tongue was flabby, and on its surface were leucomatous patches. At the back of the tongue, just in front of the V formed by the circumvallate papillæ, was a little cluster of raised warts, of a medium-brown colour, each of the size of a pea, well defined. These growths felt sore; they had existed for more than two years, and had been frequently cauterised, without benefit. The patient smoked about half an ounce of shag a day. He was ordered a mixture containing:

Liq. Hyd. Perch., ʒi.

Sp. Amm. Aromat., ♏xx.

Decoct. Cinchonæ. Co., ℥i.

three times a day; also a mouth-wash of chlorate of potash. Smoking to be discontinued.

After three weeks of this treatment, the brown warty growths had completely disappeared. The whole tongue was firmer and cleaner, and the leucomatous patches less marked.

The treatment was continued: all the symptoms, including the effusion into the knee, rapidly disappeared. A few leucomatous patches were left on the tongue; these, however, did not cause him uneasiness. They belonged to the smooth whitey-blue class, rather than to the raised and scaly. This is the only case of these peculiar brown warty growths which I have seen. I cannot find a description of a similar condition.

The nigrites described by Butlin in his interesting book have somewhat the same clinical features. He believes they are due to parasitic action. This case cleared up under anti-specific remedies. The leucomatous condition is very common in men who smoke freely. One frequently sees cases in which, not only the tongue, but the inside of the lips, cheeks, and in some cases even the palate, is covered with these patches. It causes no inconvenience; but men suffering from it are liable to ulceration of the parts upon slight provocation. I have a patient under my care in whom a well-defined leucomatous patch has existed on the tongue for more than twenty years without change. He formerly smoked a great deal, and the patch is on the side of the mouth in which he held his pipe. It causes him no inconvenience, and I only discovered it accidentally. He laughingly said that lots of doctors had tried to cure it, but could not, and that it was nothing, though he was formerly anxious about it.

*Painful Fissure and Ulcer of the Tongue. Pain referred to the temporal region and ear.*—T. C., æt. 48. Twenty-two years ago he was under treatment for syphilis. He was fumigated and salivated. In spite of this, he had a rash all over him, and since then has had occasional remedies. For some years his tongue had been a source of trouble to him. The patient had a pained, careworn look, and complained of intense shooting pain in the left temporal region and left ear. This had existed for some weeks, and had been rendered worse by caustics. His tongue was deeply fissured, with prominent whitish ridges between. At the left side of the tongue was a small oval superficial ulcer, which, when touched, made him wince with pain, which he referred to the regions mentioned. It was probably referred from the lingual nerve to the auriculo-temporal, both branches of the third division of the fifth nerve. He smoked about one ounce of tobacco a day, and stated that it relieved his pain. He could only speak and swallow with difficulty, and mouthed his words. He was ordered :

Liq. Hyd. Perchlor., ʒi.

Sp. Ammon. Aromat., ♏xx.

Decoct. Sarsæ Co., ℥i.

three times a day. He was also recommended to stop smoking, to wash his mouth out as frequently as possible with tepid water, particularly after each meal. The surface of the ulcer was to be dried and painted over three times a day with a solution of chromic acid, ten grains to the ounce. This relieved the pain very speedily and markedly, and in a very short time the ulcer healed. The fissures also healed, and the ridges became less prominent and less white.

After five weeks' treatment, all the symptoms had disappeared except a little leucoma. He was strongly advised to continue the medicine ; but, as is usual with these cases, he failed to attend after the prominent symptoms were relieved. He will probably return in a short time with a similar condition.

*Mucous Patches in the Mouth.*—A. B., æt. 25, a heavy drinker, with bloated plethoric face, contracted a hard sore four months before he presented himself at the hospital. He had been slightly treated, but induration and other traces of the chancre still remained. He complained only of pain in his mouth and difficulty in swallowing. On examination, the tongue, inner part of the lower lip, soft palate, uvula, and pillars of the fauces were found to be covered with typical mucous patches. The chest and abdomen were thickly covered with a papular eruption.

He was ordered one grain of grey powder in pill three times a day. The patches to be painted over three times a day with a solution of chromic acid, 10 grs.—℥i.

Under this treatment the patches rapidly disappeared; so much so, that on his next visit, a week afterwards, no traces remained. He persevered with the treatment for a month, with marked improvement of all his other symptoms, and then ceased his attendance, contrary to advice. This was in August, 1887.

In November he came up again, complaining of sore mouth. He admitted drinking hard for some weeks. On his tongue, near the tip, were two superficially excavated sores, with whitish margins, about the size of threepenny pieces. On the anterior part of the hard palate was a row of superficial ulcers, similar to those on the tongue. The rash over the body was now macular. His tongue was swollen and very tremulous. Under these circumstances he was ordered a magnesian mixture and some chromic acid paint. The week after, his symptoms were again markedly relieved, and the anti-specific course was re-commenced. But the patient again failed to attend.

These cases illustrate the value of chromic acid as a local application in mucous patches, and also in some painful ulcers. The drawback to its use is the rapidity with which the symptoms are relieved. The patients will not believe that a long course of treatment is necessary; they say they are well, and, in spite of advice, entreaties, or commands, follow their own sweet wills. I know no remedy which acts with such rapidity in these cases.

*Severe Asthma Cured by Nose Treatment.*—J. M., æt. 32, came to the hospital complaining of a rupture. His breathing was very asthmatical. While discussing the question of the radical cure of his hernia, I told him he should get his asthma treated. He looked on this as beyond remedy, as he had taken medicine without benefit. He then told me it came on about ten years previously, after working at brick-burning. During this process a quantity of sulphurous acid gas is given off, which irritates the nostrils. This roused my attention, and, before sending him to my medical colleague for treatment, I examined his nose. The mucous membrane was greatly thickened and vascular—so much so, that the inferior turbinated bones looked like marbles, and were pressing against the septum, which was also greatly thickened. The patient breathed through his mouth; his extraordinary muscles of inspiration were excessively developed, and his whole chest moved up and down in a typical emphysematous manner.

The patient complained that he was always wheezy and short of breath. This condition had existed uninterruptedly for ten years, but was aggravated by foggy weather. I should have added that his father suffered from asthma.

Feeling sure that the asthma was due to the vascular hypertrophic rhinitis, I scarified the turbinated bones and septum freely with a tenotomy knife, and encouraged the bleeding which followed. Ordered him a purge every day, boracic lotion to syringe up his nose, and boracic and eucalyptus ointment to smear over the inside after doing so. A week after the patient presented himself, remarking that "the cutting has done me a good deal of good." His breathing was markedly improved, the thickening ot the membrane less, and less vascular.

After another fortnight of the same treatment, he presented himself, remarking: "I feels new like altogether." His breathing was quite natural, the swelling of the membrane was very much less, and a distinct interval existed between the septum and turbinated bones, through which the patient could blow freely.

The treatment was continued, with improvement of the nasal hypertrophy; but soon after the patient ceased his attendance, and postponed the radical cure of his hernia to a more convenient season.

*Peculiar Inflammation, resulting from Cat-bites.*—Three cases have come under my care in the last six months, presenting rather similar clinical features. Two of the patients were bitten by cats, the third scratched her finger in opening a meat-tin.

Case 1. Man, æt. 62, while trying to separate a cat and dog, received a bite from the cat in the fleshy part of his thumb.

After a few days the part began to swell, and on the the third day after the occurrence he applied at the hospital for relief.

The whole thumb was then swollen, of a dull-red colour, which disappeared on pressure; there was no marked œdema, but a peculiar tense, glazed appearance of the skin. He complained of great pain in the part, which also shot up the arm. The top of the thumb was almost white, apparently from the tense nature of the swelling. The patient was very nervous, and complained of feeling much upset.

He was ordered a purge, and poppy-head fomentation for his thumb. The pain and other symptoms persisted, and after a few days he was ordered to poultice the part and to take a mixture of iron and quinine. This was continued for a fortnight, but relieved him very little. Though there was no evidence of suppuration, an incision was recommended, but declined by the patient. The thumb was next wrapped in lint soaked in a 1–20 solution of carbolic acid, still without relief. After a few days the patient consented to have the part incised. A free incision was made; not much blood followed, and no pus. After this the condition gradually improved, and at the end of a month the patient had regained the use of his thumb, though it remained weak.

The lack of response to treatment first drew my attention to the case. The notes were taken towards the termination, and are, therefore, not so precise as one could wish; but the main facts are correct.

Case 2. Woman, æt. 44. Came to the hospital on October 20th, 1887, complaining of a painful finger. She was a debilitated-looking woman, and suffered from bronchitis. A week before admission she was bitten on the finger by a cat. The finger presented the same swollen, tense, glazed, dull-red appearance as the last case. The pain was severe and extended up the arm, the colour disappeared on pressure, and the finger was very tender.

From previous experience, incision was recommended and consented to: "anything to relieve the pain." There was no pus in this case, and very little blood followed the incision; but improvement commenced, and continued uninterruptedly until her discharge, on November 21st. At that time the redness and glazed swelling had disappeared; she could use the finger, but it felt weak. The patient took a mixture of iron and quinine.

Case 3. M. G., æt. 31, a healthy-looking woman, complained of a painful swelling of the forefinger. This presented similar appearances to the cases already described. Ten days before admission the patient scratched her finger while opening a meat-tin; it gradually swelled and became painful.

On admission, the forefinger was swollen, glazed, tense, painful, of a dull-red colour, except at the distal phalanx, where it was almost white. At the base of the proximal phalanx, the swelling and redness were abruptly defined by a raised edge, its dull-red colour contrasting strongly with the adjoining skin. The redness disappeared on pressure, and slowly returned. The pain extended up the arm to the axilla, and was excessive. An incision was declined.

On the supposition that it was some low form of erysipelas, she was ordered 15 grains of salicylate of soda four times a day, and the finger was wrapped in lint washed with lotio hyd. perchlor., 1–500. She returned three days after, but without abatement of the symptoms. The swelling and redness had not extended. The veins of the arm on that side were distended, but not tender, and they showed no signs of phlebitis. She was then ordered a mixture of quinine and magnesia, and locally equal parts of glycerine and extract of belladonna spread on lint.

The symptoms gradually abated.

These three cases presented very similar clinical features, and pointed to a low form of inflammation. Only one of the patients (No. 2) belonged to the asthenic type, and yet, in all, the inflammation was of that class. It struck me the change might be produced by the slow development of a poison of a slight degree of intensity. In all the cases the swelling and redness were well defined, and yielded slowly to treatment. My experience of cases 1 and 2 points to early incision as the most satisfactory treatment.

*Syphilitic Mother. Eight Consecutive Miscarriages.—Anti-specific treatment—Healthy living child—Subsequent miscarriage.*

M. M., æt. 40, came under my treatment in 1884, when house-surgeon at the Hospital, for a sore on the knee, which had existed for some months. It had the suspicious appearance of a syphilitic sore, and on questioning the patient, I found she had had eight consecutive miscarriages. She was then pregnant—rather more than two months gone. She expressed a wish to have a healthy living child, and promised to persevere with the necessary treatment. She was ordered:

Liq. Hyd. Perchlor., ʒss.

Sodii Iodid, gr. ij.

Pot. Iodid., gr. ij.

Sp. Am. Aromat., ♏xx.

Decoct. Sarsæ Co., ℥j.

three times a day.

She continued this treatment, with occasional intermissions, when she took an iron and quinine mixture, until her confinement at full term. A fine, healthy child was born, and at the time the mother was in a good state of health. I advised her to bring the child up by hand, but soon after lost sight of her.

Last October she appeared in my out-patient room, with a rash on her face and the old knee trouble, and asked for some more of the same medicine. She rapidly improved under its use. She then told me that the healthy child was still living, and in good condition. About twelve months before, Oct., 1886, she had had another miscarriage, for which catastrophe she did not express much regret.

This case is of interest, as showing how the transmission of taint and tendency to abort, shown by eight consecutive miscarriages, may be combated by the persistent use of antispecifics. It also shows that when the disease has taken firm hold of the system, the course must be continued for a very long period. I have no doubt that had she persevered, her next child would have been born alive.

*Chancre of the Upper Lip.*—G. T., æt. 22, March 19th, 1887, came complaining of a sore on the lip. Eighteen months previously he had a sore on the penis, followed one month after by a rash all over the body. For this, he was treated for some months in the Newcastle-on-Tyne Infirmary. In January, 1887, he noticed a pimple on the upper lip, which gradually became large and hard. About six weeks before, he had been shaved for the first time in his life by a stranger, and had a slight cut on the upper lip.

The sore was raised, circular, about the size of a threepenny piece, with a scantily-discharging surface, and a well-defined indurated base. At the angles of the jaw were indolently enlarged hard glands. A true Hunterian chancre was diagnosed. He was ordered one grain of grey powder three times a day, and lotio nigra. The sore healed, and the induration subsided in a month; and then the patient discontinued his attendance.

Aug. 3rd, 1887. He reappeared, looking very thin and anæmic, with an indolent ulceration behind the pillars of the fauces.

Aug. 27th. He had a circular ulcer of the palate, and a deep ulceration, of a horse-shoe shape, in the pharynx; mucous patches on the lips, and a papular rash over the chest.

This time he was ordered: ol. morrhuae, ʒij, and

Liq. Hyd. Perchlor., ʒj.

Pot. Iodid., gr. iij.

Sp. Am. Aromat., ♏xx.

Decoct. Sarsæ Co., ℥j.

three times a day. He improved rapidly, but failed to attend after Oct. 5th.

On Nov. 10th he reappeared, with very extensive and deep ulceration of the pharynx. This also healed rapidly; but after Dec. 22nd he again disappeared.

This was an undoubted case of chancre of the lip, and was probably caused by infection from a dirty razor.

The hospital authorities at Newcastle-on-Tyne were communicated with, but could furnish no information about the sore on the penis. I am inclined to think he had an attack of syphilis at that time, the scar on the penis having the appearance of that left by a true chancre.

*Chancre of the Gum.*—F. S., æt. 32. Complains of a sore in the mouth.

Aug. 8th, 1887. Small ulcer on gum of upper jaw; circular, not indurated; no enlarged glands; very little discharge. He admitted having had a sore on the penis eight years before. He was now ordered some iodide of potassium and chromic acid paint locally, on the supposition that it was a late secondary ulceration.

Aug. 15th. The patient looked anæmic and ill. The sore was now larger, distinctly raised, circular, and indurated; under the jaw on that side were some indolent glands. He was ordered one grain of grey powder three times a day, and lotio nigra as a wash. Under this treatment the sore rapidly subsided; but after six weeks, he failed to attend. I have very little doubt that this sore was a primary chancre. The patient, a sailor, stated that a month or so before he noticed the sore, he had used an old tooth-brush he found lying about.

*Case of Raynaud's Disease.*—F. S., a groom, æt. 25. Oct. 31st, 1887. Came complaining of sore ears. He had suffered from rheumatism for ten years. His father died of rheumatic fever: two brothers and two sisters died young. The patient, though only 25 years of age, looked more than 40. He had a marked earthy, cachectic appearance; his forehead was low, and rather bossy; bridge of nose depressed; teeth peg-shaped and notched; fissured scars at the angles of the mouth. His heart-sounds were feeble, but no bruit was heard. The urine was acid, and contained a trace of albumen.

One week before admission, the left ear became painful, and the day after, the right ear. They then became blue and numb; the condition gradually increased to that presented on admission. On the top of the left ear was an oval patch—1 inch long, ½ inch across—black and gangrenous. The adjoining skin was purplish-yellow and very tender. A similar condition existed on the right ear, though not quite so well marked. The fingers and toes were cold, dark-bluish red, and congested. The wrists and fingers were swollen, and the wrists presented the puffy appearance seen in hard-worked horses' fetlocks, due to chronic teno-synovitis.

Raynaud describes the disease as symmetrical gangrene, due to irritation of the excito-motor centre in the cord which presides over the vaso-motor nerves. He attributes the local mischief to cramp-like contractions of the smaller arteries, due to this irritation.

In this case all the symptoms of congenital syphilis were present in a marked degree, and the gangrene appeared to be due to paralysis of the vaso-motor nerves, possibly a later stage of that described by Raynaud. The weather at the time was seasonable for October, not excessively cold.

Another similar case was under my care about the same time, but unfortunately the notes have been mislaid.

*Rheumatic Orchitis.*—J. B., æt. 33, came complaining of swollen and painful testicles. He was a married man: his wife had five healthy children by him.

There was no syphilitic, traumatic, or gonorrhœal cause, and he denied having caught cold. There was no family or acquired tendency to phthisis. Two days before admission he noticed that the left testicle was swollen and painful, and the day after, the right testicle. For the fortnight previously, he had suffered from aching pains about the body; and during the last week his hands had became swollen and painful. The right testicle was enlarged and very tender; the left markedly so, being about twice its natural size. The epididymis of both testicles was enlarged, as was also the cord. The greater part of the swelling was a soft solid, but a small quantity of fluid was found in each tunica vaginalis. Above the left testicle, and detached from it, was a tender, defined swelling, about the size of a walnut, with an elastic feel. A slight varicocele existed on both sides. From the acute onset and painful nature of the swelling, combined with the absence of gonorrhœa or traumatism, and the pain in other parts, rheumatic orchitis was diagnosed. He was ordered a purge, and 15 grains of salicylate of soda four times a day. Five days after, both testicles were smaller and less painful; the swelling above the left testicle was reduced to half the size; the fluid in the tunicæ vaginales had became absorbed; the hands were better. He continued the treatment for a fortnight, and was discharged at the end of that time. The testicles had returned to their normal size; all the fluid had disappeared; the swelling above the left testicle could not be discovered; and the veins were much smaller, and there was no pain.

Cases of rheumatic orchitis, pure and simple, are not common. Rheumatism frequently predisposes to and complicates orchitis, the exciting cause of which is generally gonorrhœa. In this case, rheumatism appeared to be the sole cause; and the rapidity with which salicylate of soda relieved the condition was surprising. He had no local treatment.

